# *Leptospira interrogans* Endostatin-Like Outer Membrane Proteins Bind Host Fibronectin, Laminin and Regulators of Complement

**DOI:** 10.1371/journal.pone.0001188

**Published:** 2007-11-14

**Authors:** Brian Stevenson, Henry A. Choy, Marija Pinne, Matthew L. Rotondi, M. Clarke Miller, Edward DeMoll, Peter Kraiczy, Anne E. Cooley, Trevor P. Creamer, Marc A. Suchard, Catherine A. Brissette, Ashutosh Verma, David A. Haake

**Affiliations:** 1 Department of Microbiology, Immunology, and Molecular Genetics, University of Kentucky College of Medicine, Lexington, Kentucky, United States of America; 2 Division of Infectious Diseases, Veterans Affairs Greater Los Angeles Health Care System, Los Angeles, California, United States of America; 3 Department of Medicine, University of California Los Angeles School of Medicine, Los Angeles, California, United States of America; 4 Department of Chemistry, University of Kentucky, Lexington, Kentucky, United States of America; 5 Institute of Medical Microbiology and Infection Control, University Hospital of Frankfurt, Frankfurt am Main, Germany; 6 Department of Molecular and Cellular Biochemistry, University of Kentucky College of Medicine, Lexington, Kentucky, United States of America; 7 Department of Biomathematics, University of California Los Angeles School of Medicine, Los Angeles, California, United States of America; 8 Department of Veterinary Sciences, University of Kentucky, Lexington, Kentucky, United States of America; Columbia University, United States of America

## Abstract

The pathogenic spirochete *Leptospira interrogans* disseminates throughout its hosts via the bloodstream, then invades and colonizes a variety of host tissues. Infectious leptospires are resistant to killing by their hosts' alternative pathway of complement-mediated killing, and interact with various host extracellular matrix (ECM) components. The LenA outer surface protein (formerly called LfhA and Lsa24) was previously shown to bind the host ECM component laminin and the complement regulators factor H and factor H-related protein-1. We now demonstrate that infectious *L. interrogans* contain five additional paralogs of *lenA*, which we designated *lenB, lenC, lenD, lenE* and *lenF*. All six genes encode domains predicted to bear structural and functional similarities with mammalian endostatins. Sequence analyses of genes from seven infectious *L. interrogans* serovars indicated development of sequence diversity through recombination and intragenic duplication. LenB was found to bind human factor H, and all of the newly-described Len proteins bound laminin. In addition, LenB, LenC, LenD, LenE and LenF all exhibited affinities for fibronectin, a distinct host extracellular matrix protein. These characteristics suggest that Len proteins together facilitate invasion and colonization of host tissues, and protect against host immune responses during mammalian infection.

## Introduction

Leptospirosis is a zoonotic disease of humans caused by the spirochete *Leptospira interrogans* and several other members of that genus [1]. The prevalence of leptospirosis in many parts of the world is due to chronic kidney infection of a wide variety of domestic, peridomestic and wild reservoir host mammals, including rodents, pigs, cattle, horses and dogs. Colonization of the renal tubules of carrier animals results in shedding of virulent leptospires in the urine. Leptospires persist in fresh water until infection of a new host occurs via the conjunctiva, breaks in the skin or by invasion of mucous membranes in the respiratory or digestive tract. A hallmark of leptospiral infection is early and widespread hematogenous dissemination manifested by fever, myalgia, conjunctivitis, meningitis, uveitis and/or jaundice. Between 5 and 10% of patients progress to the more dangerous, icteric phase of leptospirosis, which may lead to death due to acute renal failure, pulmonary hemorrhage, intracerebral hemorrhage, and multiorgan system failure [1]. Infectious *Leptospira* spp. are endemic in many tropical and temperate areas of the world, presenting health threats to inhabitants of both rural and urban areas, as well as military personnel, aid workers, and tourists.

Presumably as mechanisms that facilitate tissue invasion and colonization, pathogenic leptospires interact with a variety of host extracellular matrix (ECM) components, and some of the bacterial adhesins have been identified [reference [2]–[6] and this work]. *L. interrogans* is highly resistant to the alternative pathway of host complement activation [7]–[12], a feature that is associated with binding of factor H to the bacterial outer membrane, degradation of C3b and C3 convertase, and inhibition of membrane-attack complex formation [11], [12]. The capacities to bind host ECM and factor H are associated with virulence, as those traits are held by infectious *Leptospira* species but are lacking from non-infectious species of *Leptospira* [2], [11], [12].

A previous study which screened an *L. interrogans* expression library for proteins capable of binding host factor H identified an approximately 25 kDa outer membrane protein, designated LfhA (leptospiral factor H-binding protein A) [12]. LfhA was also found to bind human factor H-related protein 1 (FHR-1), a distinct protein whose carboxy-terminus is very similar to that of factor H [12], [13]. LfhA did not bind human factor H-like protein 1 (FHL-1), a splice variant of the same gene as factor H which consists of the first seven short consensus repeat units of factor H [12], [13].

A subsequent study by Barbosa and colleagues identified an *L. interrogans* outer membrane laminin-binding protein, which they designated Lsa24 (leptospiral surface adhesin 24kDa) [2]. Intriguingly, Lsa24 and LfhA are the same protein, indicating that this single protein is able to bind host factor H, FHR-1 and laminin.

As described in the present report, *L. interrogans* carries 5 additional genes homologous to *lfhA/lsa24*. Modeling of the predicted proteins encoded by these six paralogous genes indicated substantial similarities to mammalian endostatins. In order to unify the nomenclatures of these leptospiral genes, we renamed *lfhA/lsa24* as “*lenA*” (leptospiral endostatin-like protein A) and named the newly-described paralogs *lenB, lenC, lenD, lenE* and *lenF*. At the present time, there are no tools available to specifically mutate *L. interrogans*, so it is impossible to study protein function in situ by deletion and complementation mutagenesis. That being so, in order to better understand these genes and their roles in leptospiral pathogenesis, we characterized relationships of *len* genes among several distinct *L. interrogans* strains, performed biophysical characterization of the proteins, and examined interactions between Len and host ECM and complement-regulatory proteins.

## Results

### Paralogous L. interrogans len genes

**Figure 1:**
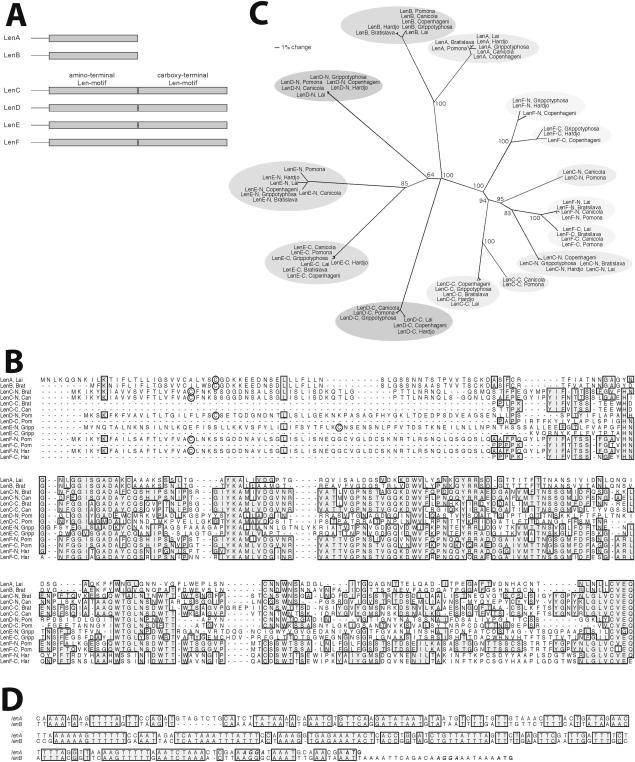
Relationships between Len proteins and genes. (A) Schematic of Len proteins, with individual Len-motifs indicated by grey rectangles. LenC, LenD, LenE and LenF each consist of 2 Len-motifs, bridged by proline-rich linkers. (B) Alignment of predicted amino acid sequences of representative proteins: serovar Lai LenA (LenA, Lai), serovar Bratislava LenB (LenB, Brat), serovar Bratislava LenC-1 (LenC, Brat), serovar Canicola LenC-2 (LenC, Can), serovar Pomona LenD (LenD, Pom), serovar Grippotyphosa LenE (LenE, Gripp), serovar Pomona LenF-1 (LenF,. Pom), and serovar Hardjo LenF-1 (LenF, Har). Sequences of the proteins possessing two Len-motifs (LenC, LenD, LenE and LenF) were divided in the middle, after the well-conserved internal CVEQ sequence, to permit alignment of each Len-motif, and the amino- and carboxy-terminal Len-motifs are indicated by “-N” and “-C”, respectively. An alignment of these same proteins, undivided, is presented in Figure S1. Identical amino acids found in the majority of proteins are boxed and shaded. Cysteine residues that may serve as amino-terminal lipidation sites are circled. (C) Unrooted phylogenetic tree of predicted amino acid sequences of each identified Len protein. Bootstrap values of each major node are indicated. (D) Alignment of sequences located 5′ of *lenA* and *lenB* genes. Identical nucleotides are boxed and shaded.

In a previous study [12], members of our laboratories demonstrated that the *lenA* (*lfhA*) genes of serovar Pomona strain JEN4 and serovar Lai strain 56601 encode a factor H/FHR-1 binding protein. Sequence analyses of additional *L. interrogans* strains from five other serovars indicated that all contain *lenA* loci. The *lenA* gene sequences of serovars Lai, Copenhageni, Grippotyphosa and Hardjo are all highly conserved and are predicted to encode membrane-associated lipoproteins (Fig. 1) [14], [15]. The *lenA* loci of serovars Pomona, Bratislava and Canicola are nearly identical to those of the aforementioned serovars, but include mutations that would preclude their synthesis of intact lipoproteins.

The sequenced genomes of *L. interrogans* serovar Copenhageni strain Fiocruz L1-130 and serovar Lai strain 56601 both contain an additional locus with size and sequence similar to *lenA*, which we named *lenB* (Table 1, Fig. 1). All other *L. interrogans* strains we examined also contain a *lenB* locus, bordered by sequences similar to those of serovars Copenhageni and Lai. The *lenB* loci of serovars Bratislava and Hardjo each encode a protein with a lipoprotein leader polypeptide and a cleavage/lipidation sequence (Fig. 1) [14], [15]. However, all other analyzed serovars lack the initial 19 codons of *lenB*, as well as a substantial portion of upstream DNA, and appear to be defective genes. Thus, only serovars Bratislava and Hardjo are predicted to produce membrane-associated LenB lipoproteins. The 5′ non-coding regions of the Bratislava and Hardjo *lenB* loci contain extensive homologies to the 5′ non-coding regions of *lenA* loci (Fig. 1D).

**Table 1:** ORF numbers of *len* genes contained in completed genomes of *L. interrogans*.

Gene name	*L. interrogans* serovar Lai strain 56601 (GenBank/TIGR) a	*L. interrogans* serovar Copenhageni strain Fiocruz L1-130
*lenA*	LA0695/LA0695	LIC12906
*lenB*	LA3103/LA3102	LIC10997
*lenC*	LA0563/LA0563	LIC13006
*lenD*	LA1433/LA1433	LIC12315
*lenE*	LA4324/LA4323	LIC13467
*lenF*	LA4073/LA4072	LIC13248

a GenBank and TIGR assigned different identifying numbers to many ORFs of *L. interrogans* serovar Lai strain 56601.

Four additional paralogs of *lenA* and *lenB* were identified in the genome sequences of Copenhageni Fiocruz L1-130 and Lai 56601, which we named *lenC, lenD, lenE*, and *lenF* (Table 1, Fig. 1). The latter four genes were sequenced in their entirety from five additional *L. interrogans* isolates. Each of the four genes in all seven strains was intact and predicted to encode a protein of approximately 50kDa. The nearly two-fold increase in size compared to LenA and LenB was accounted for by the presence of two motifs that each resemble LenA and LenB, which we refer to as “Len-motifs” (Fig. 1A). Sequence relatedness between paired Len-motifs of *lenC, lenD, lenE*, and *lenF* indicates that the motif duplications arose intragenically. In all four predicted proteins, the Len-motifs are separated by a proline-rich, 5–20 amino acid linker sequence (Fig. 1B and Fig. S1).

Phylogenetic analyses indicated that, in most cases, sequence differences between homologous *len* loci of the different *L. interrogans* serovars occurred after the evolutionary diversification of the loci themselves. As examples, all *lenA* genes are very closely related to each other, as are also the identified alleles of *lenB, lenD* and *lenE* (Fig. 1C). However, the *lenC* gene sequences of serovars Bratislava, Copenhageni, Grippotyphosa, Hardjo and Lai are very similar to each other, but are divergent from the *lenC* genes of serovars Canicola and Pomona, which are also nearly identical to each other (Fig. 1B and C). In the below-described studies of Len functions, we refer to the former group as *lenC*-1, and the latter as *lenC*-2. The *lenF* genes also fell into two distinct clades, designated *lenF-1* and *lenF-2* (Fig. 1B and C). Computational analyses indicated a stronger likelihood of a recombination event having given rise to the two distinct *lenF* groups, as opposed to a shared history, with a log_10_ Bayes factor of approximately 8 (a log_10_ Bayes factor >2 is considered to be strong evidence [16]). The *lenF-1* group of serovars Bratislava, Canicola, Lai and Pomona apparently arose from a recombination event in which both *len-motif*s of a primordial *lenF* gene were replaced by motifs of the *lenC* lineage. The other three examined serovars, Copenhageni, Grippotyphosa and Hardjo, form the distinct *lenF-2* group, which does not exhibit evidence of such a recombination event. The *lenF-1* recombination event appears to have involved only the *len-motif*s of that group: the sequences of the *lenF-1* and *lenF-2* groups are virtually identical in both the 5′ coding and noncoding regions, and the 3′ noncoding region, and those regions differ in sequence composition from analogous regions of the purported *lenC* donor (Fig. 1B and data not shown). These data indicate that proteins of the LenF-1 group are likely to be mosaics of two parental lineages: *lenF*-derived in the amino-terminal region of the protein, including the signal peptide, and *lenC*-derived in the two Len-motifs.

Comparisons of *len* gene sequences also revealed evidence of DNA transfer between leptospires. For example, serovars Pomona and Lai contain very similar *lenF-1* genes, but Pomona contains a *lenC-2* locus while Lai contains a distinct, *lenC-1* variant of that gene (Fig. 1C).

**Figure 2:**
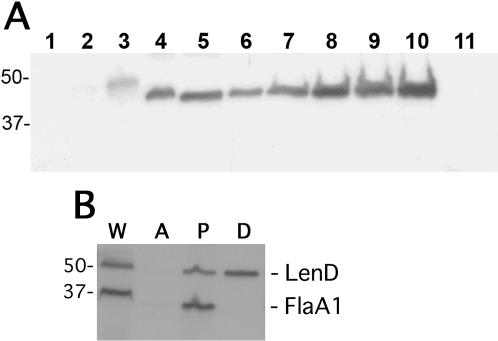
(A) Infectious *Leptospira* species produce a protein similar to *L. interrogans* LenD. Immunoblot of bacterial lysates using polyclonal rabbit antiserum raised against recombinant *L. interrogans* serovar Pomona LenD. Lanes 1, 2, and 3 contained 0.5 µg of recombinant LenA, LenC, and LenD, respectively, demonstrating the specificity of the antiserum. Lanes 4-11 contained whole-cell lysates from several different species of *Leptospira*: (4) *L. interrogans* serovar Copenhageni strain Fiocruz L1-130; (5) *L. interrogans* serovar Pomona strain PO-01; (6) *L. kirschneri*; (7) *L. noguchii*; (8) *L. santarosai*; (9) *L. borgpetersenii*; (10) *L. weilii*; (11) *L. biflexa*. Locations of molecular size standards (in kDa) are shown to the left. Note that the recombinant LenD protein includes a fusion partner and is not lipidated, so exhibits a different mobility than do the native proteins. (B) LenD localizes to the *L. interrogans* outer membrane, as assessed by Triton X-114 extraction. *L. interrogans* serovar Copenhageni LI-130 whole-cell lysate (lane W), the aqueous fraction (lane A, containing periplasmic proteins), the insoluble pellet (lane P, containing cytoplasmic cylinders and intact bacteria) and the detergent fraction (lane D, containing outer membrane proteins) were subjected to immunoblot using polyclonal rabbit antisera raised against LenD and FlaA1, a component of the inner membrane-associated endoflagella.

Alignments of non-coding regions preceding *lenC, lenD, lenE*, and *lenF* loci did not reveal any obvious sequence similarities with each other or with *lenA* or *lenB* (data not shown). That result suggests that the *lenC, lenD, lenE*, and *lenF* operons might be regulated independently of each other and *lenA/lenB*, since each operon's 5′ non-coding DNAs could bind distinct regulatory factors. Supporting that hypothesis, cultured *L. interrogans* serovar Copenhageni Fiocruz L1-130 produced detectable levels of LenD protein, but not of LenC, LenE or LenF (Fig. 2 and data not shown), and *len* gene transcript levels are affected differently when *L. interrogans* is cultivated under varying conditions [17], [18].

Southern blot analyses indicated that *lenA* genes are carried by all examined species of pathogenic leptospires, but absent from non-infectious *Leptospira* species [12]. Further evidence of widespread maintenance of *len* genes among infectious *Leptospira* species was provided by immunoblot analyses. Polyclonal rabbit antiserum raised against recombinant LenD recognized LenD but none of the other *L. interrogans* Len paralogs (Fig. 2A and data not shown). That antiserum identified an approximately 50 kDa protein in whole-cell lysates of all examined pathogenic *Leptospira* species, including strains of *L. kirschneri*, *L. noguchii*, *L. santarosai*, *L. borgpetersenii*, and *L. weilii* (Fig. 2A). No such protein was detected in the non-pathogenic species *L. biflexa*.

### Cellular localization and biophysical characterization of Len proteins

LenA is an outer membrane protein [2], [12]. All Len proteins bear at least some hallmarks of being lipoproteins, with LenA, LenB, LenC, LenD and LenF having high probabilities of being so according to analyses using the spirochete-specific lipoprotein algorithm SpLip [14], [15]. Using the above-described anti-LenD antiserum, cellular localization of that protein was assessed by Triton X-114 detergent solubilization and phase partitioning of live leptospires. The reliability of this technique has been validated by comparisons with results obtained by sucrose density gradient isolation of outer membrane vesicles [19]. This method yields three fractions: a detergent fraction consisting of outer membrane components, an aqueous fraction consisting of periplasmic contents, and a pellet consisting of inner membrane-associated components, cytoplasmic contents, and undisrupted cells [20]. LenD was found in the detergent fraction (Fig. 2B). Control analysis of the endoflagellar sheath protein FlaA1 (which is attached to the inner membrane) confirmed that the detergent phase was not contaminated with inner membrane components (Fig. 2B). Presence of LenD in the pellet fraction is typical of leptospiral outer membrane proteins [21]–[23], and is indicative of incomplete Triton X-114 extraction. These results indicate that LenD is also an outer membrane protein.

The LenC, LenD, LenE and LenF proteins appear to be fused dimers of LenA/LenB-like proteins. This suggested to us that LenA and LenB might function as dimers, with each dimer being the equivalent of a single LenC, LenD, LenE or LenF protein. To explore that possibility, we examined whether or not recombinant LenA forms dimers. However, HPLC through a size-exclusion column yielded a single peak, with a calculated molecular mass of 24.2 kDa (data not shown), comparable to the calculated molecular mass of 24.4 kDa for the recombinant LenA monomer.

**Figure 3:**
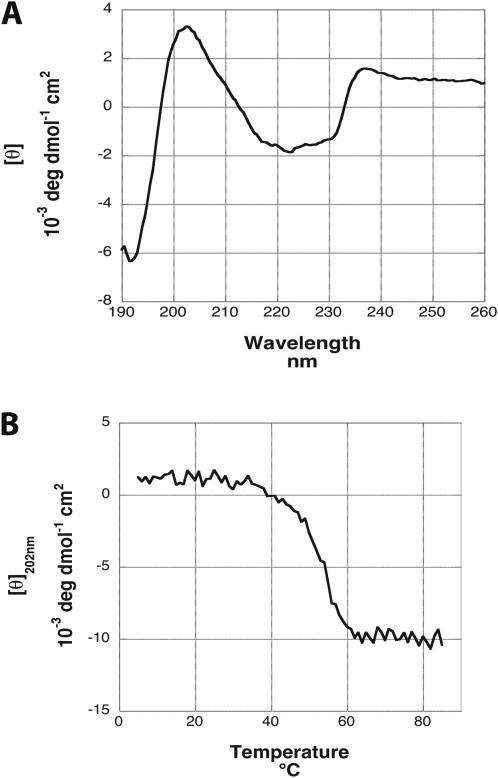
Biophysical analysis of recombinant LenA. (A) Circular dichroism spectrum of recombinant LenA. Deconvolution indicated this protein to consist of 36% beta-sheet, 23% turns, and 41% unordered/other structures. (B) Melting analysis of recombinant LenA, indicating a relatively stable protein with a melting point of 53°C (±2°C).

Circular dichroism (CD) analysis of recombinant LenA indicated that it is composed of 36% β-sheet, 23% turns, and 41% unstructured/other, with no evidence of any α-helices (Fig. 3A). These data are in line with previous CD analyses indicating that LenA contains β-sheets [2]. Recombinant LenA was found to be a relatively stable protein, with a melting point of 53°C (±2°C) (Fig. 3B). Due to the limited solubility of the other recombinant Len paralogs, those proteins could not be analyzed by these biophysical techniques. PHYRE modeling of the predicated amino acid sequences of all Len proteins indicated moderate to strong probabilities (ranging between 25 and 60% estimated precision) for each Len-motif folding into a structure similar to those of mammalian endostatins, which are derived from the carboxy-termini of collagens XVIII and XV [24]–[26]. Among other functions, endostatins bind various ECM components, including laminin, [25], [26]. We did not detect sequence or predicted structural similarities between Len proteins and any other known adhesins.

### Functional characterization of Len paralogs

**Figure 4:**
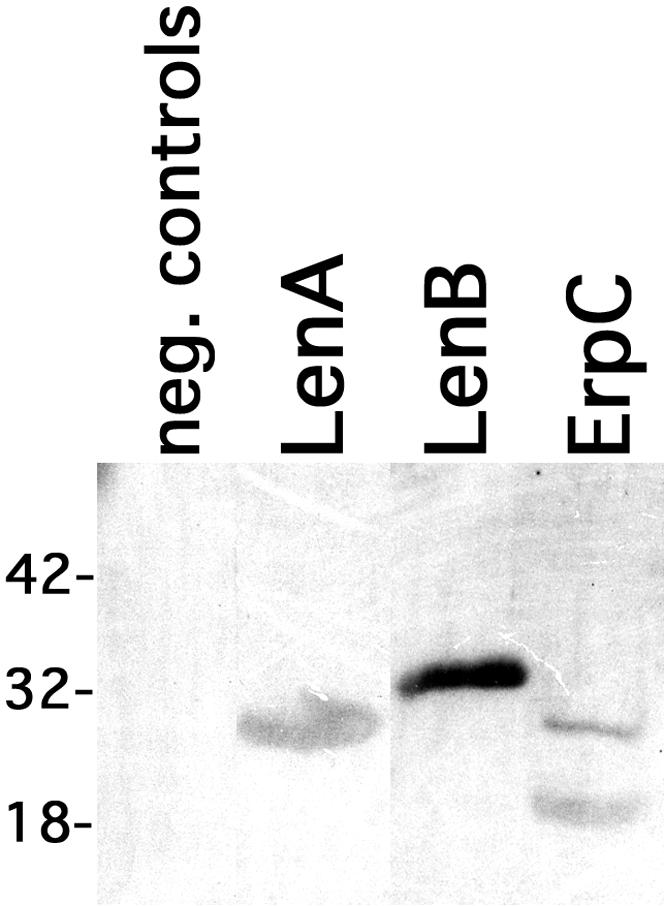
LenB binds human factor H. Ligand affinity blot analyses of recombinant LenB, with recombinant LenA and *B. burgdorferi* ErpC proteins included as positive controls [71]. Carbonic anhydrase, soybean trypsin inhibitor and lysozyme were loaded onto the same lane and served as both negative controls and molecular mass standards. Positions of those standards are indicated to the left of the panel (in kDa).

Through use of both affinity blot analyses and surface plasmon resonance, members of our laboratories previously demonstrated that LenA binds host complement factor H [12]. Ligand affinity blot analyses of LenB indicated that it, too, can bind human factor H (Fig. 4). However, none of the other Len proteins exhibited binding of factor H, as assessed by ligand affinity blot.

**Figure 5:**
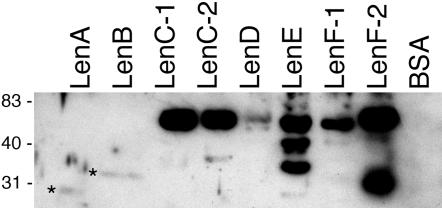
Ligand affinity blot analyses of recombinant Len proteins with purified laminin. Asterisks indicate positions of relatively weak signals corresponding to binding of laminin by LenA and LenB. Smaller bands seen in some lanes correspond with protein degradation products, indicating that at least some of the larger Len proteins can bind laminin even when partially truncated. Bovine serum albumin (BSA) was included in all blots as a negative control. Positions of molecular mass standards are indicated to the left (in kDa).

A previous study indicated that recombinant LenA (Lsa24) bound laminin [2]. We therefore examined whether or not the five newly-identified *L. interrogans* proteins shared that property. Each recombinant protein was solubilized in SDS buffer, subjected to SDS-PAGE and transferred to nitrocellulose membranes, then examined for ability to bind soluble laminin. All the recombinant Len proteins bound laminin, although LenC-1, LenC-2, LenE, LenF-1 and LenF-2 consistently yielded the strongest affinity blot signals, with LenD, LenB and then LenA exhibiting progressively weaker relative binding of laminin (Fig. 5). No laminin binding by control protein BSA was detected, even with extended film exposure times, demonstrating that binding of laminin by the Len proteins was specific.

**Figure 6:**
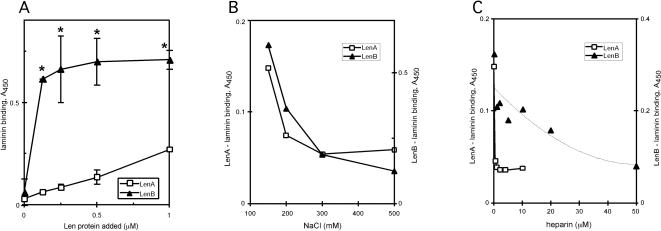
ELISA results of LenA and LenB binding to laminin. Soluble recombinant LenA and LenB were each examined for binding to 1 µg immobilized laminin. (A) Saturable laminin binding by LenB compared to the weaker binding by LenA. Average of two independent experiments (bars equal 1 standard deviation), as representative of additional assays performed with different preparations of Len proteins. Significant differences (P<0.05) between LenA and LenB are indicated by asterisks The mean K_d_ for LenB binding is 118 +/− 39 nM (n = 4). (B) Laminin binding by LenA and LenB is dependent on ionic strength. (C) Heparin competes with LenA and LenB for laminin binding. The activity of 1 µM LenA was measured with heparin added to the binding buffer. The higher avidity of LenB was challenged by preincubation of laminin with heparin prior to adding 0.25 µM LenB plus varying concentrations of heparin.

Binding by soluble LenA and LenB was examined further by ELISA, using laminin immobilized in microtiter wells as the target ligand. LenB showed saturable binding (K_d_ = 118 +/− 39 nM , mean and standard deviation from four independent experiments), while LenA exhibited significantly weaker activity (Fig. 6A). The other recombinant Len proteins were insoluble in buffers compatible with ELISA, preventing their characterization using that technique.

Ligand affinity blot analyses showed adverse effects on laminin binding by all recombinant Len proteins when the ionic strength of the TBS-T buffer was increased (data not shown). The dependence on ionic interactions for laminin binding by LenA and LenB was examined by ELISA in the presence of increasing concentrations of NaCl. When compared to laminin-binding activity in buffer containing 150 mM NaCl, binding to LenA was reduced 49 and 60% by 200 mM and 500 mM NaCl, respectively, while binding to LenB was inhibited 40 and 79% by 200 mM and 500 mM NaCl, respectively (Fig. 6B).

Since laminin may interact with charged moieties through its “heparin-binding” sites [27], we examined the ability of heparin to compete with Len proteins for binding to laminin. One µM heparin reduced the ability of 1 µM LenA to bind laminin to 26% of the no-heparin control (Fig. 6C). One µM and 4 µM heparin inhibited the laminin-binding activity of 1 µM LenB to 75% and 55% of the no-heparin control (data not shown). A more pronounced inhibition, to only 25% of the no-heparin control, was observed when immobilized laminin was preincubated with 50 µM heparin followed by ELISA using 0.25 µM LenB in the presence of 50 µM heparin (Fig. 6C).

**Figure 7:**
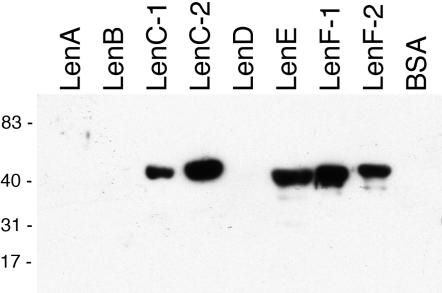
Ligand affinity blot analyses of recombinant Len proteins using purified fibronectin. Prolonged film exposures indicated binding of fibronectin by LenB and LenD, but increased background signal made it impossible to produce a clear figure (see Fig. S2). No indication of LenA binding to fibronectin was observed at any exposure. Bovine serum albumin (BSA) was included in all blots as a negative control. Positions of molecular mass standards are indicated to the left (in kDa).

LenA was previously reported to bind weakly to fibronectin [2]. Ligand affinity blot analyses were therefore used to explore the abilities of the other Len proteins to bind soluble fibronectin. Strong binding signals were obtained for LenC-1, LenC-2, LenE, LenF-1, and LenF-2 (Fig. 7). Weaker signals from LenB and LenD were visible following prolonged film exposure times (see Fig. S2), suggesting lower avidities of those two proteins relative to the other five leptospiral proteins. By this technique, no signals were detected from LenA or from the negative control protein, BSA.

**Figure 8:**
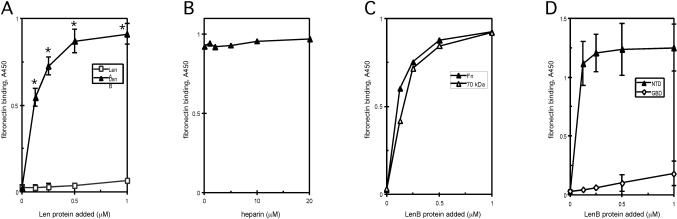
ELISA results of LenA and LenB interactions with fibronectin or its proteolytic fragments. (A) Saturable binding of LenB to intact fibronectin, with calculated K_d_ 106±8 nM (means and standard deviations from three experiments). Significant differences (P<0.05) between LenA and LenB binding are indicated with asterisks. (B) Binding of fibronectin by LenB is not affected by heparin. Fibronectin was preincubated with heparin, then binding by 1 µM LenB was analyzed in the presence of additional heparin. (C) Interaction with the 70-kDa N-terminal fragment of fibronectin (70 kDa) can account for complete LenB binding to intact fibronectin (Fn). (D) High avidity binding of LenB to the NTD of fibronectin, with calculated K_d_ 69.5 nM (means and ranges from two experiments). LenB did not appreciably bind the fibronectin GBD.

Fibronectin binding was further examined by ELISA using soluble recombinant LenA and LenB. LenB exhibited strong, saturable binding (K_d_ = 106±8 nM, from three experiments, Fig. 8A). Our analyses indicated relatively weak interactions between LenA and fibronectin, much as was previously described [2], and was not studied further. In contrast to laminin binding, heparin did not detectably affect LenB-fibronectin interaction (Fig. 8B). The binding activity of 0.25 µM LenB remained intact even in the presence of 50 µM heparin (data not shown). Assays with proteolytic fragments of fibronectin indicated that the N-terminal 70 kDa could account for all of the binding by LenB observed with intact fibronectin (Fig. 8C). This fragment comprises the type I repeat modules in fibronectin, which can be divided into two functional domains, the N-terminal domain (NTD; available as a 30kDa fragment) and the adjacent gelatin-binding domain (GBD; a 45 kDa proteolytic fragment) [28]. LenB interacted only with the 30 kDa NTD, with K_d_ = 69.5 nM±0.7 nM (Fig. 8D). *L. interrogans* LigB, which binds both the NTD and the GBD [3], served as a positive control for GBD binding (data not shown).

## Discussion

*L. interrogans* is an invasive extracellular pathogen, capable of disseminating through its hosts' bloodstream to the kidneys and other organs, then colonizing those tissues. To do so requires that the bacterium evade complement-mediated killing and adhere to host cell surfaces and/or extracellular matrices, especially epithelial and endothelial basement membranes. We have extended upon earlier studies by demonstrating that infectious strains of *L. interrogans* encode up to six distinct paralogous proteins with affinities for host fibronectin, laminin, factor H and/or FHR-1. Differences in ligand binding were apparent among the Len paralogs: recombinant LenC, LenE and LenF exhibited appreciably greater affinities for laminin and fibronectin than did the other paralogs, LenB bound both those ligands more tightly than did LenA, and only LenA and LenB were demonstrated to bind host factor H. Such diversification of function is frequently observed in other organisms following gene duplication events [29].

The 5′ noncoding regions of the intact *lenA* and *lenB* genes showed extensive similarities, but that pair of loci and the *lenC, lenD, lenE* and *lenF* loci all differed considerably in their 5′ noncoding regions, suggesting that transcription of each is likely to be governed by a distinct regulatory mechanism. This study and previous array studies support of that hypothesis, with culture temperature having opposite effects upon transcription of *lenD* and *lenE* [17], osmolarity of culture medium significantly affecting only *lenD* [30], and only LenD being produced at detectable levels by *L. interrogans* serovar Copenhageni Fiocruz L1-130 when cultured in EMJH medium (this work). Many other leptospiral genes also exhibit differences in expression during mammalian infection, growth in the external environment, or when cultured in media of various compositions or temperatures [17], [18], [30]–[33]. Diversification of gene regulatory elements is also a frequent occurrence among paralogous gene families [34].

The roles of Len protein interactions with host proteins during infection processes remain to be determined. Laminins are important components of basement membranes, and fibronectin is a major component of both ECM and serum, so binding those host proteins could facilitate interactions directly with ECM or serve as a bridge for adherence to cell surfaces [27], [28], [35]. Factor H is a regulator of the alternative pathway of complement activation, and adheres to eukaryotic cells through a variety of specific and non-specific receptors [13], [36]–[39]. Binding of factor H by *L. interrogans* may therefore help protect the bacterium from killing by complement and/or serve as a bridge to facilitate adherence to host tissues [40]. The functions of FHR-1 and other factor H-related proteins are poorly understood, but appear to include control of complement activation, cellular adherence and other functions [13], [41]–[45]. One feature held in common by all the identified host ligands of Len proteins is their affinities for heparin/heparan sulfate. In our studies, binding of both LenA and LenB to laminin were inhibited by heparin. LenB was determined to bind the amino-terminal domain of fibronectin, which contains heparin-binding sites. However, the inability of heparin to inhibit fibronectin-binding by LenB indicates that either fibronectin has a much higher affinity for LenB than for heparin or LenB contains one binding site for laminin and a second site that binds fibronectin. LenA binds both factor H and FHR-1, which contain highly similar heparin-binding regions, but LenA does not bind FHL-1, which lacks that heparin binding domain [12], [13]. Many additional vertebrate proteins are known to bind heparin/heparan sulfate [46], and we are continuing to investigate whether such proteins may also be ligands for leptospiral Len proteins.

Gene duplication events probably gave rise to the six *len* paralogous genes and the paired *len*-motifs of the larger genes. A different type of recombination event led to the *lenF-1* clade, with *len*-motifs of the *lenC* lineage replacing the homologous sequences of a primordial *lenF* gene. Intriguingly, the new *lenF-1* lineage retained its ancestral *lenF*-like leader polypeptide and the flanking non-coding DNA sequences. Phylogenetic mosaicism has previously been observed for another leptospiral gene, which encodes the OmpL1 porin [47].

In conclusion, infectious *L. interrogans* encode six paralogous Len proteins that can interact with host laminin, fibronectin, and/or complement regulatory proteins. All six members of the Len family share structural and functional characteristics with mammalian endostatins, fragments of collagens XVIII and XV which bind laminin and other cell surface and tissue proteins. The apparently widespread distribution of *len* genes among virulent leptospires, their presence in multiple copies in *L. interrogans* genomes, and their absence from non-pathogenic *Leptospira* species, suggest that Len proteins perform important roles during pathogenesis and have provided a selective advantage during mammalian infection. Generation of *len* sequence diversity occurred early during leptospiral evolution through genetic drift and recombination between *len* genes, prior to the development of distinct antigenic serovars. Diversity within the paralogous Len family appears to have resulted in functional differences, which may facilitate colonization of multiple niches and hosts. While site-specific genetic manipulation of *L. interrogans* is currently impossible, further in vitro studies on functions of the Len proteins and analyses of their expression during infection will continue to increase our understanding of the mechanisms of host tissue interactions and complement evasion employed by this pathogen.

## Materials and Methods

### Bacterial strains and culture conditions

Infectious *L. interrogans* serovar Copenhageni strain Fiocruz L1-130 [48] was obtained from Albert Ko (Gonçalo Moniz Research Center, Oswaldo Cruz Foundation, Brazilian Ministry of Health, Salvador, Bahia, Brazil). Infectious *L. interrogans* serovar Lai strain 56601 [49] was obtained from Mathieu Picardeau (Pasteur Institute, Paris, France). Infectious *L. interrogans* serovar Pomona type kennewicki strain JEN4 was isolated from an infected horse in Kentucky, USA [32]. *L. interrogans* reference organisms Pomona strain Pomona, Copenhageni strain M 20, Canicola strain Hond Utrech IV, Grippotyphosa strain Andaman, Hardjo strain Hardjoprajitno, and Bratislava strain Jez Bratislava were obtained from Michael Donahue (Livestock Disease Diagnostic Center, University of Kentucky, Lexington, KY) and were of undetermined infectivity. Infectious *L. interrogans* serovar Pomona strain PO-01, *L. kirschneri* serovar Grippotyphosa strain RM52, *L. noguchii* serovar Proechymis strain LT796, *L. santarosai* serovar Bakeri strain LT79, *L. borgpetersenii* serovar Hardjo strain HB-15B7/93U, and the non-infectious saprophyte *L. biflexa* serovar Patoc strain Patoc I were obtained from the National Animal Disease Center, Agricultural Research Service, United States Department of Agriculture (Ames, IA). Infectious *L. weilii* strain Ecochallenge was isolated from the blood of an infected human who participated in Eco-Challenge 2000 held in the Malaysian Borneo [50]. All leptospires were grown at 30°C in Ellinghausen-McCullough-Johnson-Harris (EMJH) broth containing 1% rabbit serum [51].

### Analyses of *L. interrogans len* gene and predicted Len protein sequences

*lenA* and paralogous genes of the previously sequenced *L. interrogans* serovars Lai and Copenhageni [48], [49] were identified by BLAST-P analyses of GenBank (http://www.ncbi.nlm.nih.gov/blast) and the Institute for Genomic Research (TIGR) Comprehensive Microbial Resource database (http://tigrblast.tigr.org/cmr-blast).

Genomic DNA from *L. interrogans* strains was isolated from 5 ml cultures, as previously described [52]. DNA segments that included each *len* locus were PCR amplified using rTaq DNA polymerase (Takara, Otsu, Japan) and 25 cycles of 94°C for 1 min, 55°C for 1 min and 72°C for 2 min. Oligonucleotide primers utilized (Table 2) were complementary to conserved sequences located 5′ and 3′ of the *len* genes of serovar Pomona strain JEN4, serovar Canicola strain Fiocruz L1-130, and serovar Lai strain 56601 [12], [48], [49]. Amplicons were cloned into pCR2.1 (Invitrogen, Carlsbad, CA) and both strands sequenced completely (Davis Sequencing, Davis, CA).

**Table 2:** Oligonucleotide primers used to amplify and clone *len* loci.

Locus	Oligonucleotide name	Sequence relative to amplified locus	Sequence (5′ to 3′)
*lenA*	LFH-2	5′	TTA GTC GGT AAT AGA GTT TTA GCG
	LFH-11	3′	ACA ATC TTC CAA AGA TCC TAA CG
*lenB*	3102-1	5′	TTT TTG ATG GCT GCA GAA ATG GGG
	3102-2	3′	AAC TTA CTG TTC TAC ACA GAG TAG
	3102-4	3′	TTC TAC TAT TAG CCT GAA AGC CTG
*lenC*	563-1	5′	ATT ACG CCA AAC TAA CGT TAA TCG
	563-4	3′	TTA CTC GTC ATT GAA AAA AGG TTG
*lenD*	1433-1	5′	AAA TAT CTA AGT TAC CGT CGC TCG
	1433-2	3′	TCA TCA TCT ACG CAA AGA ATT GCG
*lenE*	4323-1	5′	ACA GAA GTC TAT CTT CAG AAT GAG
	4323-2	3′	ATG AGA TTC AAA ATA ATC GAT CGG
*lenF*	4072-1	5′	TTG AAA AAA ATG AAA TCC AGC CTG
	4072-4	3′	TTT TCG AAC GGG CCT AAG ATT GAG

DNA and protein alignments were performed using Clustal X [53]. Phylogenies were reconstructed both by the neighbor joining method under default settings of amino acid substitution using PAUP* version 4.0b10 software [54] and by Bayesian inference under the Hasegawa, Kishino, Yano nucleotide substitution model [55] and a relaxed molecular clock [56] using BEAST 1.4 software. In the former case, bootstrapping provided measures of clade credibility [57]. To estimate the Bayes factor in favor of a recombination event [58] giving rise to the *lenF-1* group over the alternative hypothesis in which *lenF-1* and *lenF-2* sharing a common history, an unconstrained tree topology model and a model in which *lenF-1* and *lenF-2* sequences are constrained to be monophyletic were fit. From each model, the marginal likelihood was estimated using the harmonic mean estimator [59] and the Bayes factor found by taking the ratio. If two hypotheses are equally likely a priori, then the Bayes factor is the relative posterior probabilities of the competing hypotheses; a log_10_ Bayes factor >2 is generally taken as strong evidence [16].

The spirochete-specific lipoprotein algorithm SpLip [15] was used to determine the probabilities that each *len* gene encodes a lipoprotein. This algorithm is a hybrid weight matrix approach using 28 experimentally verified spirochetal lipoproteins in the training set. All lipoproteins contain a hydrophobic amino-terminal leader polypeptide, followed by variable 3–4 amino acid “lipobox”, then a cysteine residue [14], [60]. During processing of the pre-protein to the mature lipoprotein, the leader polypeptide is removed and lipid moieties attached to the cysteine. The −1 position relative to the cysteine is the most constrained position in the lipobox, and is typically populated by a small, uncharged amino acid. The four most frequently-occurring residues at the −1 position in leptospiral lipoproteins are serine, asparagine, glycine and alanine (listed in descending order of frequency).

### Recombinant Len proteins and antibodies

A polyhistidine-tagged recombinant LenA protein, based upon the *lenA* gene of serovar Lai, was previously described [12]. Additional polyhistidine-tagged recombinant proteins were produced using pET200 (Invitrogen). Recombinant LenB was produced from the gene of serovar Bratislava, one of the two serovars identified as having a complete *lenB* ORF. Recombinant proteins LenC-1 and LenC-2 were produced from the genes of serovars Bratislava and Canicola, respectively, representatives of the two *lenC* allele groups. As the *lenD* and *lenE* genes each form a tight phylogenetic cluster (Fig. 1C), serovars Pomona and Grippotyphosa were chosen at random for production of recombinant proteins LenD and LenE, respectively. The *lenF* genes of serovars Pomona and Hardjo served as templates for recombinant proteins LenF-1 and LenF-2, respectively, representatives of the two allele groups of that gene. Recombinant proteins formed insoluble inclusion bodies when produced in *Escherichia coli*, and so were purified in the presence of 8M urea using MagneHis nickel conjugated magnetic beads (Promega, Madison, WI). As a final step in purification, recombinant proteins were dialyzed against PBS using 10 kDa Slide-a-Lyzer cassettes (Pierce, Rockford, IL). Each of the new recombinant proteins precipitated out of solution during dialysis, and all except LenB remained insoluble unless 8M urea was included in solvent. LenB dissolved into PBS after 2–3 days incubation at 4°C. Concentrations of the insoluble LenC-1, LenC-2, LenD, LenE, LenF-1 and LenF-2 recombinant proteins were determined by SDS-PAGE of homogeneous suspensions and Coomassie brilliant blue staining alongside protein standards of known concentration.

Polyclonal rabbit antisera directed against each Len protein were produced by Animal Pharm Services (Healdsburg, CA), using one round of their standard vaccination procedure (www.animalpharmservices.com). Briefly, approximately 2 mg of recombinant protein was suspended into PBS by vigorous mixing, then divided into 6 equal aliquots. A New Zealand White rabbit was injected 6 times over a 6 week period, then serum collected 1 week after the final injection.

### Triton X-114 extraction

Cultures of *L. interrogans* serovar Copenhageni strain LI-130 (approximately (10^9^ cells/ml) were fractionated using Triton X-114 [20]. Bacteria were pelleted by centrifugation, washed in PBS containing 5 mM MgCl_2_, then extracted with 0.5% protein-grade Triton X-114 (Calbiochem), 150 mM NaCl, 10 mM Tris, pH 8, and 2 mM EDTA, at 4°C. Insoluble material was pelleted by centrifugation at 16000×g for 10 min. After centrifugation, 1 M CaCl_2_ was added to the supernatant, to a final concentration of 20 mM. Phase separation was performed by warming the supernatant to 37°C, and subjecting it to centrifugation for 10 min at 2000×g. The detergent and aqueous phases were separated, and proteins precipitated with acetone. Proteins were then separated by SDS-PAGE, blotted onto PVDF membranes and probed with polyclonal sera raised against leptospiral Len proteins or the flagellar component FlaA1 [61].

### Size fractionation chromatography

The ability of recombinant LenA protein to form multimers was determined by gel filtration chromatography, using a Waters 600 pump and controller equipped with a Waters 996 photodiode array UV/Vis detector (Waters, Milford, MA). A Superdex 75 10/300 GL column (GE Healthcare) was prepared with a mobile phase consisting of 200 mM NaCl, 50 mM Tris-HCl (pH = 7.5), 1% (vol/vol) glycerol. The column was run with a flow rate of 0.20 ml per min. The elution of each standard was determined by monitoring A_280_. A calibration curve was created using an MW-GF-70 low molecular weight calibration kit (Sigma-Aldrich), and the void volume, V_0_, was determined by injection of 200 µl of 1 mg/ml blue dextran in elution buffer with 5% glycerol. The remaining protein standards: bovine lung aprotinin (6.5 kDa), horse heart cytochrome C (12.4 kDa), bovine carbonic anhydrase (29 kDa), and bovine serum albumin (66 kDa), were individually prepared in elution buffer with 5% glycerol to total concentrations of 0.3 mg/ml. The molecular mass calibration curve was generated by plotting the log (molecular mass) vs. V_e_/V° [62]. A 200 µl sample of recombinant LenA (approximately 0.2 mg/ml) was then injected and its elution compared to the established curve.

### Protein structure analyses

Circular dichroism (CD) spectra were collected using a J-810 spectrapolarimeter equipped with a Peltier heating block (Jasco, Easton, MD). A 1mm path length cuvette was employed, with reported spectra being the average of four scans taken at scan rates of 20 nm/min. Melting scans were performed at a scan rate of 1°C/min, recording the ellipticity at 202 nm which is the wavelength at which the largest change in ellipticity was observed. Protein concentrations were determined using the method of Brandts and Kaplan [63]. Absorbance was measured in a 1.0 cm path length cuvette in a DU 640B spectrophotometer (Beckman-Coulter, Fullerton, CA). Secondary structure analysis of the CD spectra was performed using DICHROWEB (http://www.cryst.bbk.ac.uk/cdweb/html/home.html) [64]. Reported secondary structure contents are averages of those calculated using the SELCON3 [65], [66], ContinLL [67], [68], and CDSSTR [69], [70] analysis programs.

Folding probabilities of Len proteins were determined using Protein Homology/analogY Recognition Engine (PHYRE) (http://www.sbg.bio.ic.ac.uk/∼phyre).

### Ligand-binding assays

Aliquots (1 µg) of each recombinant Len protein and negative control protein BSA were subjected to SDS-PAGE, then transferred to nitrocellulose membranes. Care was taken not to overheat recombinant proteins prior to gel loading, as incubation in boiling water for longer than 15 s tended to irreversibly interfere with ligand binding. Interactions between Len proteins and purified human factor H were examined as previously described [12]. For analyses of laminin and fibronectin binding, membranes were blocked with 5% BSA in Tris-buffered saline-Tween 20 (TBS-T; 20 mM Tris, 150 mM NaCl, 0.05% Tween 20 [pH 7.5]), then incubated for 1 h at room temperature with either murine laminin or human fibronectin (both from Sigma-Aldrich) at concentrations of 20 µg/ml in TBS-T. Following extensive washing with TBS-T, membranes were incubated for 1 h at room temperature with rabbit polyclonal antibodies specific for either murine laminin (diluted 1∶5000) or human fibronectin (diluted 1∶1000) (both from Sigma-Aldrich). Finally, the membranes were washed with TBS-T and incubated for 1 h at room temperature with horseradish peroxide-conjugated protein A (GE Healthcare). Bound antibodies were detected using SuperSignal West Pico enhanced chemiluminescence substrate (Pierce).

Only recombinant LenA and LenB were soluble in the absence of urea (see above). Binding of host proteins by these soluble recombinant Len proteins was further measured using ELISA-based techniques, as described previously [3]. Immobilized target ligands included murine laminin, human plasma fibronectin, proteolytic fragments of fibronectin (70-kDa N-terminal fragment, the 30-kDa amino-terminal domain [NTD], and the 45-kDa gelatin-binding domain [GBD]) (all from Sigma-Aldrich), and human factor H (Calbiochem). In experiments testing the effect of ionic strength on Len-laminin interactions, additional NaCl was included in the PBS-based binding buffer. For heparin-competition assays, porcine heparin (Sigma-Aldrich) was added to the binding buffer along with the tested recombinant Len protein. In some experiments, heparin was also added to the laminin- or fibronectin-coated wells 1 h prior to the Len proteins. Results were reported as absorbance at 450 nm for the activity of horseradish peroxidase conjugated to a goat antibody (Novagen) against a monoclonal antibody for polyhistidine (Novagen). K_d_ values were calculated as the concentration of Len protein giving half-maximal binding. Means of independent experiments with equal variance were compared with Student's t-test and alpha at 0.05.

### Accession numbers

The new *L. interrogans* DNA sequences described in this work have been deposited in GenBank and given the following accession numbers. Serovar Pomona type kennewicki strain JEN4 *lenB, lenC, lenD, lenE* and *lenF*: EF606888 through EF606892; serovar Pomona strain Pomona *lenA*: EF606893; serovar Copenhageni strain M 20 *lenA, lenB, lenC, lenD, lenE* and *lenF*: EF606894 through EF606899; serovar Bratislava strain Jez Bratislava *lenA, lenB, lenC, lenE* and *lenF*: EF632554 through EF632558; serovar Canicola strain Hond Utrech IV *lenA, lenB, lenC, lenD, lenE* and *lenF*: EF611235 through EF611240; serovar Grippotyphosa strain Andaman *lenA, lenB, lenC, lenD, lenE* and *lenF*: EF999884 through EF999889; and serovar Hardjo strain Hardjoprajitno *lenA, lenB, lenC, lenD, lenE* and *lenF*: EF999890 through EF999895.

## Supporting Information

Figure S1 Alignment of predicted amino acid sequences of representative proteins: serovar Lai LenA (LenA, Lai), serovar Bratislava LenB (LenB, Brat), serovar Bratislava LenC-1 (LenC, Brat), serovar Canicola LenC-2 (LenC, Can), serovar Pomona LenD (LenD, Pom), serovar Grippotyphosa LenE (LenE, Gripp), serovar Pomona LenF-1 (LenF,. Pom), and serovar Hardjo LenF-1 (LenF, Har). An alignment of these same proteins, with the proteins having two Len-motifs divided after the well-conserved internal CVEQ sequence, is presented in Figure 1. Identical amino acids found in the majority of proteins are boxed and shaded. Cysteine residues that may serve as amino-terminal lipidation sites are circled.
(133.57 MB TIF)

Figure S2 Extended film exposure of ligand affinity blot analyses of recombinant Len proteins with purified fibronectin. Signals corresponding with fibronectin bound to LenB and LenD are indicated by asterisks above each band. No indication of LenA binding to fibronectin was observed at any exposure. Bovine serum albumin (BSA) was included as a negative control. Positions of molecular mass standards are indicated to the left (in kDa).
(0.30 MB TIF)
