# Supplementary figures and images for: Leptospira interrogans Endostatin-Like Outer Membrane Proteins Bind Host Fibronectin, Laminin and Regulators of Complement
Source: PLoS One. 2007 Nov 14;2(11):e1188. doi: 10.1371/journal.pone.0001188 (PMC2063517; doi:10.1371/journal.pone.0001188)

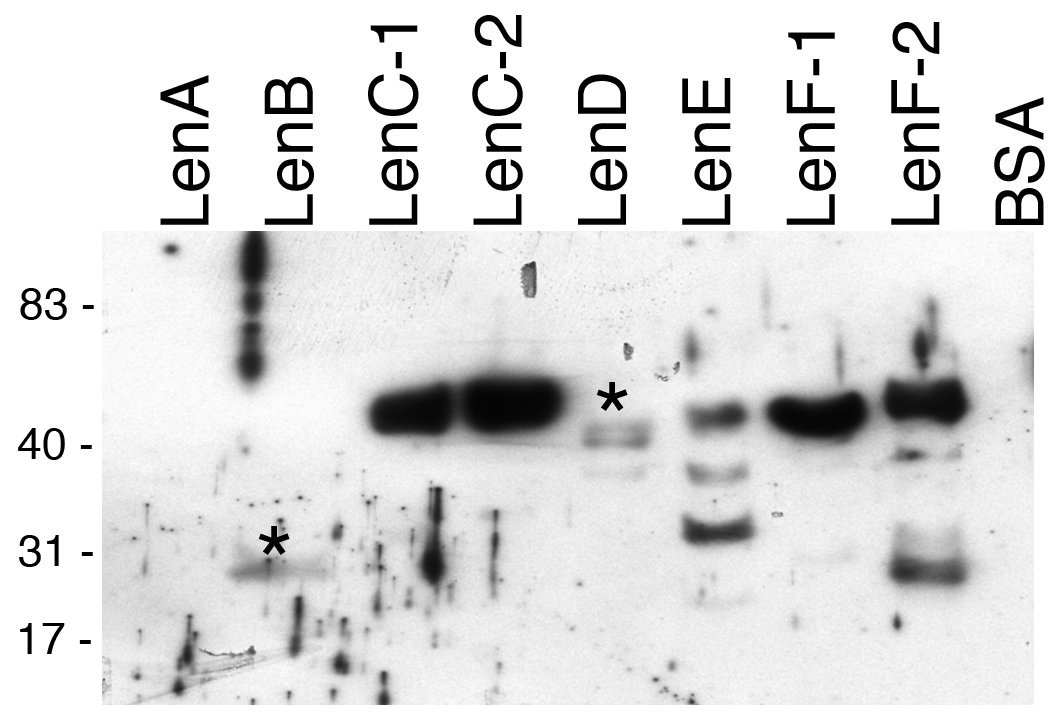

Supplement: Figure S2 — Extended film exposure of ligand affinity blot analyses of recombinant Len proteins with purified fibronectin. Signals corresponding with fibronectin bound to LenB and LenD are indicated by asterisks above each band. No indication of LenA binding to fibronectin was observed at any exposure. Bovine serum albumin (BSA) was included as a negative control. Positions of molecular mass standards are indicated to the left (in kDa). (0.30 MB TIF) [file pone.0001188.s002.tif]
